# Peripheral Vasculitis, Intermediate Uveitis and Interferon Use in Multiple Sclerosis

**DOI:** 10.4274/tjo.35555

**Published:** 2016-01-05

**Authors:** Şeref Kinyas, Haluk Esgin

**Affiliations:** 1 Trakya University Faculty of Medicine, Department of Ophthalmology, Edirne, Turkey

**Keywords:** Multiple sclerosis, intermediate uveitis, peripheral vasculitis, Interferon

## Abstract

Multiple sclerosis (MS) is a chronic inflammatory demyelinating disease of the central nervous system. A 40-year-old female patient with a 12-year history of MS was admitted to our clinic with blurred vision and floaters in her right eye for about 1 month. Here, we share the findings and the management of intermediate uveitis and retinal periphlebitis in an MS case being treated with interferon beta-1a for 7 years.

## INTRODUCTION

The most common ocular sign in multiple sclerosis (MS) patients is optic neuritis, although uveitis may also be present at a rate varying between 0.4 and 26.9%. Uveitis is more common among women between 20 and 50 years of age; although chronic, the long-term visual prognosis is usually good. Intermediate uveitis is the most common form of uveitis seen in MS and is characterized by vitreous condensation and snowball-like structures in the pars plana and peripheral retina. Retinal periphlebitis may occur in 5-36% of cases, presenting with exudation and vascular sheathing due to the accumulation of inflammatory cells.^1^ The association between MS and retinal vein sheathing was first described by Rucker^2 in 1944. Rucker2^ reported that retinal vasculitis was mild, temporary and asymptomatic, especially in patients with only periphlebitis, without the presence of choroiditis.

Medications such as interferon beta-1a (IFN-β-1a) (intramuscular and subcutaneous forms), IFN-β-1b, glatiramer acetate, natalizumab and mitoxantrone are currently used in the long-term treatment of MS and have a positive impact on the course of the disease. In clinical studies, IFN-β has been shown to decrease the frequency of attacks and slow disease progression. Interferons also have immune system suppressing, anti-inflammatory effects. IFN-β prevents proinflammatory cytokine production and antigen presentation by inhibiting T cell activation. It also shows an immunomodulatory effect by preventing the migration of lymphocytes into the central nervous system, thus preventing demyelination and decreasing the frequency and severity of MS attacks.^3^

## CASE REPORT

**Figure 1:**
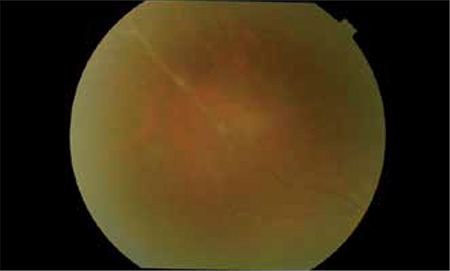
Perivenous sheathing and exudation in the peripheral retina of the right eye

**Figure 2:**
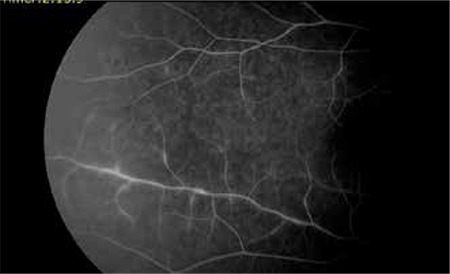
Hyperfluorescence due to with segmental minimal vascular leakage in the right eye

A 40-year-old female patient presented to our clinic with complaints of blurred vision and floaters in the right eye for 1 month. She had a 12-year history of MS and had been treated with intramuscular IFN-β-1a (Avonex® 30 µg [6 million IU]) once a week for the previous 7 years. Her visual acuity was measured as 1.0 in both eyes using the Snellen chart. Her pupils were isochoric and a relative afferent pupillary defect was observed in her right eye. In the Farnsworth-Munsell 40 Hue test, her color vision was 18/40 in the right eye and 26/40 in the left eye. Slit-lamp examination of the anterior segment of both eyes was normal and no inflammation was observed. Intraocular pressure was 15 mmHg in the right eye and 16 mmHg in the left as measured by Goldmann applanation tonometry. Ophthalmoscopic examination revealed vitritis (+1), widespread perivenous sheathing in the peripheral retina, and exudation in both eyes, but more pronounced in the right eye (Figure 1). On fundus fluorescein angiography (FFA) of the right eye, staining around the major arc and hyperflorescence due to minimal vascular leakage in the perivenous segments were observed; FFA in the left eye revealed staining of the vessel walls of the inferior temporal vein branches (Figure 2).

**Figure 3:**
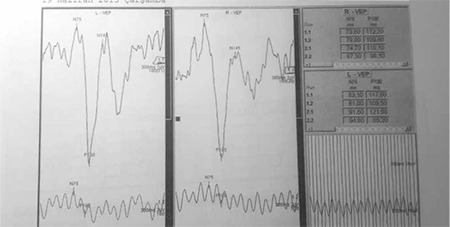
Pattern visual-evoked potential analysis revealed significant p100 latency delays in both eyes

**Figure 4:**
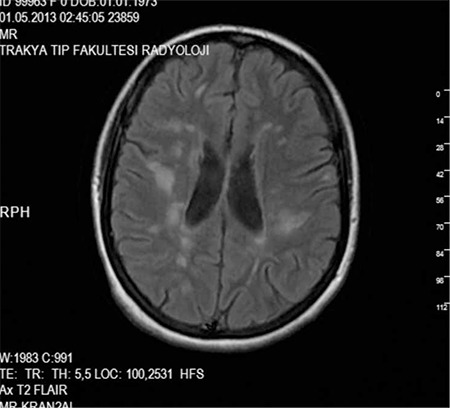
Cranial magnetic resonance imaging showing multifocal demyelinating plaques in the periventricular area

**Figure 5:**
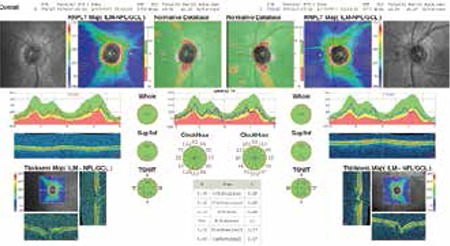
Retinal nerve fiber layer thickness at the optic disc was within normal range in both eyes

On pattern visual-evoked potential (pVEP) analysis, the p100 latency and amplitude were 112.20 ms and 12.3 µV in the right eye, 117.60 ms and 12.3 µV in the left eye, respectively (Figure 3). Localized visual field defects were observed in the right eye on automatic static perimetry (Octopus 500), while the left eye was normal. Cranial magnetic resonance imaging revealed multifocal demyelinating plaques in the periventricular area (Figure 4). The patient had perfect visual acuity (1.0) in both eyes, so she was advised to continue systemic IFN-β-1a without additional ophthalmologic treatment. She was followed for two years and was clinically stable; at last examination her vision was perfect (1.0) in both eyes. Slit-lamp examination revealed (+1) cells in the anterior vitreous of both eyes. Perivenous sheathing and snowball-like exudations were observed in the anterior quadrants of both eyes during fundoscopic examination. On follow-up FFA, persistent perivenous staining and minimal vascular leakage were observed. Macula edema was not apparent in either eye on optic coherence tomography (OCT) and the retinal nerve fiber layer at the optic disc appeared normal (Figure 5).

## DISCUSSION

MS patients may show signs of ocular inflammation such as uveitis, pars planitis or retinal vasculitis. Periphlebitis is more common in the active phase of MS, and leakage due to perivenous inflammation may be observed on FFA.^4^ Yılmaz et al.^5^ reported an MS patient with bilateral iridocyclitis and retinal periphlebitis, but they could not show perivenous leakage on FFA. In our case, the appearance of perivenous leakage on FFA suggests that the inflammation was active, although it was not severe.

In our patient, the presence of relative afferent pupillary defect in the right eye, impaired color vision in both eyes, and significantly prolonged p100 latency on pVEP were considered evidence of previous optic neuritis attack and optic nerve demyelination.

IFN decreases ocular inflammation in MS patients. It has been shown to prevent the development of macular edema by decreasing vascular leakage, especially in the posterior segment. Okada et al.^6^ demonstrated clinically and histopathologically that IFN treatment was effective in decreasing ocular inflammation in rats with experimentally induced autoimmune uveoretinitis.

There are a limited number of studies investigating treatment approaches in cases with both MS and uveitis. In a case series of 13 patients with MS and uveitis, Becker et al.^7^ reported that IFN-β-1a was effective in suppressing intraocular activity and preserving visual acuity. In a study conducted in Turkey including 8 patients with MS-related uveitis being treated with IFN-β-1a, it was reported that visual acuity was largely protected and MS attack frequency and risk of serious complications were reduced.^8^ Our case used systemic IFN-β-1a for 7 years, and we also observed that retinal periphlebitis and intermediate uveitis findings were under control, visual acuity had been protected for 2 years, and there were no signs of macular edema.

Wakefield et al.^9^ reported uveitis attacks characterized by serious visual acuity decline and cystoid macular edema in a series of 5 MS patients not receiving long-term immunomodulatory therapy. After just 7 days of treatment with high-dose (1 g/day) intravenous pulse methylprednisolone, the patients’ visual acuity improved and ocular inflammation was decreased.

It was reported that retinal vasculitis was much more severe in an MS patient using glatiramer acetate, and that it was necessary to perform panretinal photocoagulation for the regression of the retinal neovascularization and preretinal hemorrhage.^10^

IFN therapy seems to be a superior treatment option due to its anti-inflammatory effects before the development of ocular inflammation, which improves the prognosis of uveitis attacks, and also because it is safer than systemic corticosteroids in terms of side effects.

## CONCLUSION

Ocular inflammation such as intermediate uveitis and retinal periphlebitis may occur in MS. In patients using IFN, ocular inflammation may be mild and asymptomatic, and visual acuity can be protected without the need for additional ophthalmologic treatment.

## Ethics

Informed Consent: It was taken.

Peer-review: Externally peer-reviewed.
